# Analysis of cytokines in serum and bronchoalveolar lavage fluid in patients with immune-checkpoint inhibitor-associated pneumonitis: a cross-sectional case–control study

**DOI:** 10.1007/s00432-021-03750-z

**Published:** 2021-08-04

**Authors:** Benedikt Kowalski, Alan Valaperti, Pascal Bezel, Urs C. Steiner, Dieter Scholtze, Stephan Wieser, Maya Vonow-Eisenring, Andrea Widmer, Malcolm Kohler, Daniel Franzen

**Affiliations:** 1 Department of Pulmonology, University Hospital Zurich, Raemistrasse 100, 8091 Zurich, Switzerland; 2 Department of Immunology, University Hospital Zurich, Gloriastrasse 23, 8091 Zurich, Switzerland; 3 Department of Pulmonology, City Hospital Triemli, Birmensdorferstrasse 497, 8063 Zurich, Switzerland; 4 Department of Pulmonology, City Hospital Waid, Tièchestrasse 99, 8037 Zurich, Switzerland

**Keywords:** Immune-checkpoint inhibitor-associated pneumonitis, Bronchoalveolar lavage, Cytokine profile, Interleukin 6

## Abstract

**Purpose:**

Immune-checkpoint inhibitors (ICI) present a new treatment for malignancies by boosting the immune system. This has led to a variety of immune-related adverse events, including ICI-associated pneumonitis (ICIaP). Diagnosis thereof is often challenging, and its pathogenesis has not yet been fully understood. The aim of this cross-sectional case–control study was to investigate cytokines in serum and bronchoalveolar lavage fluid (BALF) expressed in patients with ICIaP compared to controls consisting of healthy individuals, patients with lung cancer and patients with interstitial lung diseases (ILD) other than ICIaP.

**Methods:**

From January 2018 until June 2019, 401 adult patients with various lung diseases were prospectively enrolled in a BALF- and serum biobank, called BALOTHEK*.* Of these, 12 patients were diagnosed with ICIaP (Pembrolizumab, Ipilimumab, or both, and Durvalumab) serving as case group. Subjects with one of three diagnosis groups from BALOTHEK, including lung cancer, ILD other than ICIaP, and healthy individuals, served as matched controls. The following 11 cytokines were simultaneously analyzed in BALF and serum of each study participant: interferon gamma, tumor necrosis factor alpha, interleukin (IL) 1b, IL-2, IL-4, IL-5, IL-6, IL-8, IL-12p70, IL-13 and IL-17A. This study was approved by the local ethic review committee (BASEC-ID 2017-02,307 and 2018-01,724).

**Results:**

Absolute number and percentage of lymphocytes in BALF of patients with ICIaP were significantly higher compared to control groups. For the investigated cytokines in BALF, a significant increase of IL-6 level was shown for patients with ICIaP compared to control groups (*p* = 0.031, adjusted for multiple comparisons).

**Conclusion:**

Cytokine profile assessed in BALF shows promising potential for facilitating diagnosis and understanding of pathophysiology of ICIaP. IL-6 may not only contribute to better understanding of pathophysiology but also herald therapeutic implications for Tocilizumab.

## Background

Both, chemo- and radiation therapy used to be the common approach for cancer treatment throughout decades. Recently, immune-checkpoint inhibitors (ICI) made of monoclonal antibodies (mAB) against receptors on T-lymphocytes have been introduced as a new therapeutic ideology in fighting cancer. Ipilimumab was the first ICI to be approved, representing a breakthrough in the treatment of metastatic melanoma (Hodi et al. 2010). Since then, clinical practice has changed (Dummer et al. 2015) and new ICIs allowed treatment of various malignancies other than melanoma. Two commonly targeted immune checkpoints are cytotoxic T-lymphocyte-associated antigen 4 (CTLA-4) and the axis programmed cell death protein 1 (PD-1) and PD-1 ligand (PD-L1). Instead of repeated exposure to cytotoxic agents in traditional chemotherapy, ICIs boost the immune system by “inhibiting the inhibition” and, thus, helping it to tackle neoplasia effectively (Kroschinsky et al. 2017). Compared to chemotherapy, the side effects of ICIs are different. Whereas adverse events from chemotherapy are partially caused by a compromised immune system, those caused by immunotherapy are mainly due to immune reinforcement. Thus, autoimmunity and excessive inflammatory responses referred to as immune-related adverse events (irAE) may be induced, leading to gastrointestinal, cutaneous, endocrinal and pulmonary manifestations, among others (Chuzi et al. 2017). Pneumonitis is an uncommon, but potentially life threatening irAE. The reported incidence for pneumonitis is 1.3–11% for ICI monotherapy (Abdel-Rahman and Fouad 2016) and 6.6% for combination therapy of Ipilimumab with Pembrolizumab or Nivolumab (Nishino et al. 2016a).

The clinical presentation of ICI-associated pneumonitis (ICIaP) is non-specific and heterogeneous, ranging from asymptomatic, only radiological manifestations to mild or moderate symptoms with cough and dyspnea, and, eventually, to very severe cases of acute respiratory distress syndrome (ARDS) (Franzen et al. 2013; Naidoo et al. 2017; Rashdan et al. 2018). While computed tomography (CT) is indispensable for diagnosis, imaging of pneumonitis is also unspecific and includes various patterns of interstitial lung disease (ILD), such as organizing pneumonia (OP), non-specific interstitial pneumonia (NSIP), hypersensitivity pneumonitis (HP), acute interstitial pneumonia (AIP) and usual interstitial pneumonia (UIP) (Nishino et al. 2016b; Porcu et al. 2019; Rashdan et al. 2018). Median onset has been observed at 2.5 months after initiation of ICI therapy, with a time window ranging between 2 and 24 months (Chuzi et al. 2017; Rashdan et al. 2018). All the above-mentioned factors may contribute to delayed diagnosis of ICIaP, while pathogenesis has not yet been fully understood.

Since ICIaP is thought to result from immune driven over-reaction, changes in cytokines as immunomodulatory proteins produced by immune cells among others can be expected. While a recent study (Lim et al. 2019) successfully identified 11 cytokines in serum to predict irAE, data about cytokines expressed in pneumonitis are scarce. However, cytokines measured only in serum might be misleading in determining type and degree of inflammation in a specific organ. Various studies suggest organ-specific sample collection as the best method for exact and targeted assessment (Corcoran et al. 2013; Hosoki et al. 2015; Kreiner et al. 2010; Qazi et al. 2011; Ricker et al. 2011). Flexible bronchoscopy is an established diagnostic tool for a broad range of pulmonary diseases, since it enables minimally invasive biopsies at low risk for various techniques including bronchoalveolar lavage (BAL) (Ernst et al. 2003; Kumar and Gupta, 2015; Rivera et al. 2013). Therefore, BAL appears suitable to provide more detailed information on the lung tissue by obtaining bronchoalveolar lavage fluid (BALF).

The aim of this cross-sectional case–control study was to investigate cytokines in serum and BALF expressed in patients with ICIaP compared to control groups consisting of healthy individuals, patients with lung cancer and patients with ILD other than ICIaP. To the best of our knowledge, there is only one study (Wang et al. 2020) investigating cytokine expression in BALF in patients with ICIaP.

## Materials and methods

### Patients

The present study is part of a prospective multicenter study, which aims to establish a biobank (“*BALOTHEK*”) using blood serum and BALF for the research of various lung diseases. The samples were acquired from patients in whom BAL was performed for purpose of routine clinical evaluation. Enrolled patients were retrospectively clustered in five groups according to clinical and radiological presentation, confirmed by histology: lung cancer, sarcoidosis, ILD, drug-related pneumonitis and healthy controls. The latter group consisted of individuals who underwent bronchoscopy for assessment of chronic cough with a normal chest computed tomography (CT) finding (i.e. absence of consolidation, ground-glass opacity, nodule, mass, or interstitial changes) and without evidence of lung disease during a follow-up time of 6 months and normal pulmonary function test. Patients were excluded in case of precedent lung transplantation, general patient vulnerability such as emergencies or pregnancies and errors in sampling or processing of the samples, e.g. BAL to processing time exceeding 60 min (Valaperti et al. 2019).

From January 2018 until June 2019, a total of 401 adult patients were enrolled in *BALOTHEK*. Simultaneously, 240 patients were treated with ICIs at the Departments of Dermatology and Medical Oncology from University Hospital Zurich because of various malignancies. Of these 240 patients, 16 developed typical symptoms (i.e. cough, fever, dyspnea) and CT findings (i.e. COP, NSIP, HP, AIP) suggestive for ICIaP. After conducting BAL, however, in four patients, an alternative diagnosis other than ICIaP had been made (one patient with Melphalan-induced pulmonary toxicity, two patients with acute bronchitis and one patient with chronic cough of unknown origin). Thus, the remaining 12 patients were eventually included with the diagnosis of ICIaP confirmed by BAL. From these patients, BALF could be harvested for purpose of *BALOTHEK* and for the present study, respectively. In addition, 3 groups with 12 subjects each of *BALOTHEK* matched according to gender and age (range ± 10 years) were used as control groups and clustered as healthy subjects, patients with lung cancer and patients with ILD other than ICIaP.

### Blood specimens and processing

All blood samples were collected by nurses proficient in blood drawing as part of the routinely performed pre-interventional peripheral venous access. For differential blood count and whole blood count, 10 ml BD Vacutainer K2E tubes (EDTA, Plus Blood Collection Tubes, Becton Dickinson, Plymouth, UK) were used. To gain serum samples, whole blood was collected in 10 ml BD Vacutainer Clot Activator Tube (CAT, Plus Blood Collection Tubes, Becton Dickinson, Plymouth, UK) and centrifuged at 3500 rounds per minute (rpm) at room temperature. Thereafter, the supernatant was aliquoted and eventually stored at − 80 °C for later analyses, according to Valaperti et al. (2019). Once thawed for analysis, the samples were not frozen again.

### Bronchoscopy, BAL and processing of BALF

Bronchoscopy was performed in moderate sedation with propofol using Olympus (Tokyo, Japan) flexible bronchoscopes (190 series). BALF was obtained conforming to official recommendations (Baughman 2007; Rennard et al. 1998) by instillation of isotonic saline solution in four times 50 ml portions into the wedged pulmonary segment that showed the most prominent finding in the most recent chest CT. Through gentle suction of the same syringe that injected the solution, BALF was yielded and pooled in a collection tube. The recovered BALF was quantitatively expressed in absolute values (ml) and in percent of the instilled volume. Thereafter, BALF was extracted from the collection tube and distributed to designated tubes, absent any further substances such as anticoagulants and preservatives for routine cytological and microbiological analyses and for purpose of the study. BALF quality was deemed inadequate if the sample showed less than 10 macrophages per field, less than 2 million cells in total, increased number of epithelial cells, exceeding the macrophages, had purulent appearance, increased number of erythrocytes due to iatrogenic traumatic procedure or was already degenerated until the processing. Inferior BAL quality led to study exclusion. For processing of the study samples, BALF was centrifuged at 1′000 rpm at room temperature. The supernatant was aliquoted and stored at − 80 °C in accordance to (Valaperti et al. 2019). Once thawed for analysis, the samples were not frozen again. The routinely performed analysis of BALF for cell differentiation was performed by ADVIA 2120i (Siemens Healthcare AG, Zurich, Switzerland) via peroxidase staining. Cell differentiation included cell count, macrophages, lymphocytes, neutrophils, eosinophils, mast cells, and plasma cells.

### Cytokine analysis

A Milliplex MAP kit (human high-sensitivity T-cell magnetic bead panel) customized by Merk Millipore (Darmstadt, Germany) was used to analyze cytokines applying MAGPIX system (Luminex Corporation, Austin, TX, USA). The array contained the following 11 cytokines: interferone-gamma (IFN-γ), interleukin (IL)-1B, IL-2, IL-4, IL-5, IL-6, IL-8, IL-12p70, IL-13, IL-17A, and tumor necrosis factor alpha (TNF-α). This selection of cytokines based on several publications (Kondo 1999; Lim et al. 2019; Matsuno 2012; Ohnishi et al. 2003; Schoenfeld et al. 2019) investigating inflammatory biomarkers in drug-induced pneumonitis as well as specifically ICIaP. The preparation of standards was composed of serial dilution 1:4 of each stock standard to generate seven standard concentrations which were used to create a five-parameter logistic curve-fit standard curve with the xPONENT software (Luminex Corporation, Austin, TX, USA). Before quantifying cytokines, the high sensitivity bead panel was successfully validated and calibrated, showing a correct standard curve for each cytokine. Cytokines were determined in BALF as well as in serum.

### Statistical analysis

**Fig. 1:**
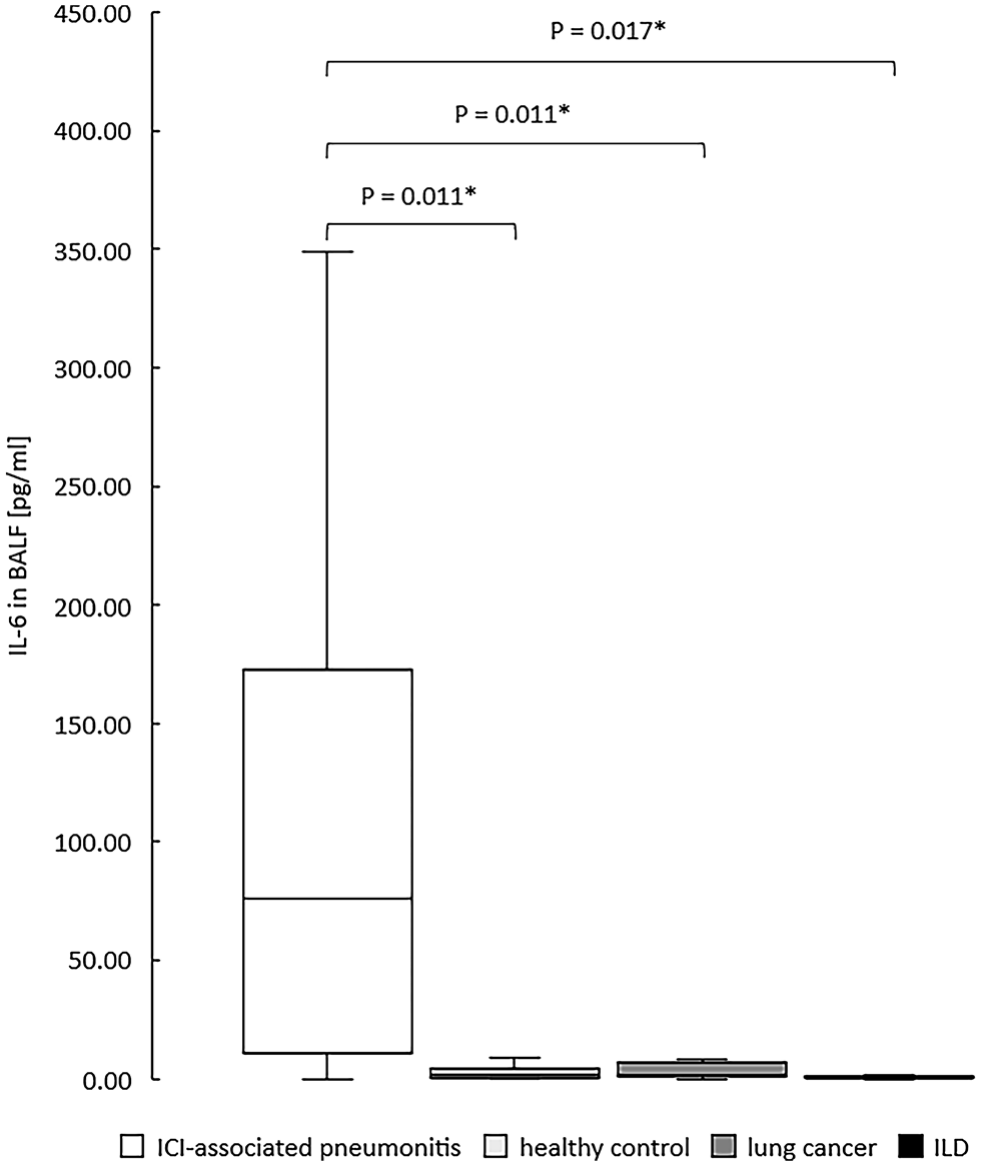
IL-6 in bronchoalveolar lavage fluid over four diagnosis groups. *p < 0.05, adjusted for multiple comparisons using the Bonferroni correction method. *ICI* immune-checkpoint inhibitor, *ILD* interstitial lung disease

Continuous data are reported as median ± interquartile range (IQR) or as mean ± standard deviation (SD), as appropriate. Normal distribution was tested using the Shapiro–Wilk test. To express comparisons between groups, Chi-squared test or Fisher’s exact test was used for categorical variables and Kruskal–Wallis test or Median test were used for continuous variables. The Bonferroni correction was used to adjust p values for multiple comparisons to avoid the risk of a type I error. P values of less than 0.05 were considered to be statistically significant and were based on two-sided hypothesis. All analyses were conducted using IBM SPSS Statistics for Windows, Version 26.0 (IBM Corporation, Armonk, NY, USA) and R Core Team, 2013; R version 4.0.3 (2020-10-10). The box plot in Fig. 1 was created with Microsoft Excel, Version 16.0 (Microsoft Corporation, Redmond, WA, USA).

## Results

Totally, 48 patients were included. From one patient with ICIaP and from one patient with ILD other than ICIaP, BALF could not be yielded due to bronchial collapse, and from one patient in the healthy control group, the serum sample was lost. Baseline characteristics and bronchoscopy data are summarized in Table 1. Specific characteristics of ICIaP population are shown in Table 2.

**Table 1:** Baseline characteristics of study population

	ICIaP	Healthy control	ILD	Lung cancer	*p* value
Number	12 (25.0)	12 (25.0)	12 (25.0)	12 (25.0)	
Demographics					
Female	5 (41.7)	4 (33.3)	5 (41.7)	5 (41.7)	0.967
Age	70.6 (61.5–76.1)	73.8 (61.6–75.3)	65.0 (53.0–73.0)	71.5 (62.3–75.3)	0.471
Smoking					
Never smoker	3 (25.0)	6 (50.0)	4 (33.3)	3 (25.0)	0.520
Current smoker	1 (8.3)	1 (8.3)	2 (16.7)	0 (0.0)	0.486
Ex-smoker	8 (66.7)	5 (41.7)	5 (41.7)	9 (75.5)	0.284
Pack years	10.0 (0.0–40.0)	3 (0.0–13.8)	8.5 (0.0–48.8)	26.0 (2.5–51.3)	0.258
Medical history					
Metastatic melanoma	7 (58.3)	0	0	0	
NSCLC (Adenocarcinoma)	5 (41.7)	0	0	9 (75.0)	
NSCLC (Squamous cell carcinoma)	0	0	0	2 (16.7)	
SCLC	0	0	0	1 (8.3)	
Pulmonary sarcoidosis	0	0	7 (58.3)	0	
NSIP	0	0	3 (25.0)	0	
RB-ILD	0	0	2 (16.7)	0	
Bronchoscopy data					
BALF in [ml]	195.5 (15.1)	191.7 (28.9)	191.7 (19.5)	195.8 (14.4)	0.910
BALF ex [ml]	74.1 (21.3)	98.6 (32.4)	87.9 (26.8)	64.6 (27.5)	0.036*
BALF recovery [%]	38.2 (11.4)	52.2 (15.8)	46.6 (15.5)	33.5 (15.2)	0.020*
BALF cell count [cells/µl]	230.1 (201.0)	104.6 (80.8)	114.7 (103.2)	41.4 (35.6)	0.023*

Data are presented as *n* (%), median (IQR) or mean (SD), as appropriate

*ICIaP* immune-checkpoint inhibitor-associated pneumonitis, *ILD* interstitial lung disease, *BALF* bronchoalveolar lavage fluid, *NSCLC* non-small-cell lung carcinoma, *NSIP* non-specific interstitial pneumonia, *SCLC* Small cell lung cancer, *RB-ILD* Respiratory bronchiolitis interstitial lung disease

*p < 0.05, adjusted for multiple comparisons using the Bonferroni correction method

**Table 2:** Characteristics of ICIaP group

Administered ICI	
Pembrolizumab	6 (50.0)
Nivolumab	2 (16.7)
Durvalumab	1 (8.3)
Ipilimumab + Pembrolizumab	3 (25.0)
Severity of ICIaP	
Grade 1	1 (8.3)
Grade 2	7 (58.3)
Grade 3	3 (25.0)
Grade 4	1 (8.3)
Concomitant medication	
Radiotherapy	2 (16.7)
Chemotherapy	3 (25.0)
Steroids	5 (41.7)
Antibiotics	5 (41.7)
None	4 (33.3)

Data are presented as *n* (%)

*ICIaP* immune-checkpoint inhibitor-associated pneumonitis, *ICI* immune-checkpoint inhibitor

From the group with ICIaP, seven subjects were treated for metastatic melanoma (58.3%) and five subjects for adenocarcinoma of the lung (41.7%). Most patients in the ILD group were diagnosed with pulmonary sarcoidosis (58.3%), whereas adenocarcinoma of the lung presented the majority in the lung cancer group (75.0%). The absolute volume of recovered BALF in patients with ICIaP was 74.1 ± 21.3 ml, referring to a recovery rate of 38.2 ± 11.4%. BALF volume in healthy individuals was significantly higher compared to other groups (*p* = 0.036), whereas BAL cell count was higher in patients with ICIaP (*p* = 0.023). Severity of ICIaP was graded based on the publication by Chuzi et al. (2017) and revealed grade 2 ICIaP for majority of patients. Most patients received concomitant medication, either as causal treatment of malignancy or for provisional diagnosis.

Absolute cell count and differentiation in serum and in BALF are summarized in Tables 3 and 4, respectively.

**Table 3:** Cell count and differentiation in serum

	ICIaP	Healthy control	ILD	Lung cancer	*p* value
CRP [mg/l]	42.5 (80.1)	4.3 (37.0)	14.4 (27.7)	6.4 (11.3)	0.002*
Leukocytes [G/l]	7.0 (2.5)	6.4 (2.7)	7.1 (3.7)	7.9 (2.7)	0.742
Neutrophils [G/l]	4.8 (2.2)	3.9 (2.4)	5.2 (3.6)	5.5 (2.5)	0.620
Monocytes [G/l]	0.7 (0.3)	0.6 (0.2)	0.5 (0.3)	0.6 (0.3)	0.555
Eosinophils [G/l]	0.1 (0.2)	0.1 (0.2)	0.1 (0.2)	0.1 (0.1)	0.989
Basophils [G/l]	0.0 (0.0)	0.0 (0.0)	0.0 (0.0)	0.0 (0.0)	0.149
Lymphocytes [G/l]	1.1 (0.4)	1.5 (0.6)	1.2 (0.6)	1.6 (0.7)	0.103
Neutrophils [%]	73.3 (9.4)	64.6 (10.5)	62.2 (24.6)	68.4 (10.0)	0.290
Monocytes [%]	9.3 (5.5)	10.4 (2.7)	7.4 (4.4)	8.1 (2.5)	0.685
Eosinophils [%]	1.9 (3.2)	2.2 (1.4)	1.9 (1.9)	1.7 (1.3)	0.953
Basophils [%]	0.3 (0.3)	0.5 (0.4)	0.5 (0.3)	0.6 (0.3)	0.344
Lymphocytes [%]	14.5 (5.3)	23.1 (10.3)	18.7 (13.0)	21.2 (7.6)	0.111

Data are presented as mean (SD)

*ICIaP* Immune-checkpoint inhibitor-associated pneumonitis, *ICI* immune-checkpoint inhibitor, *ILD* interstitial lung disease, *CRP* C-reactive protein

*p < 0.05, adjusted for multiple comparisons using the Bonferroni correction method

**Table 4:** Cell count and cell differentiation in BALF

	ICIaP	Healthy control	ILD	Lung cancer	*p* value
Macrophages [/µl]	74.7 (18.4–139.0)	52.5 (40.3–113.0)	61.0 (20.8–111.5)	36.7 (15.9–57.2)	0.243
Lymphocytes [/µl]	66.9 (30.8–152.9)	4.0 (3.4–25.4)	17.4 (4.3–61.3)	1.2 (0.5–1.7)	0.001*
Neutrophils [/µl]	10.0 (4.7–14.8)	1.0 (0.6–15.3)	2.3 (0.5–10.9)	0.6 (0.2–2.2)	0.022*
Eosinophils [/µl]	0.4 (0.0–3.7)	0.3 (0.0–2.4)	0.0 (0.0–2.2)	0.0 (0.0–0.0)	0.032*
Macrophages [%]	49.5 (27.5–62.5)	88.0 (63.3–95.3)	60.0 (54.4–77.8)	95.5 (91.0–97.0)	< 0.001*
Lymphocytes [%]	45.5 (32.0–64.0)	7.3 (3.9–13.4)	24.5 (5.6–39.4)	2.0 (1.5–3.5)	0.0001*
Neutrophils [%]	5.5 (3.0–7.5)	1.5 (0.5–10.3)	3.8 (2.1–13.9)	2.5 (1.0–5.5)	0.155
Eosinophils [%]	0.8 (0.4–1.8)	1.0 (0.0–3.0)	0.0 (0.0–1.1)	0.0 (0.0–0.0)	0.198

Data are presented as median (IQR)

*ICIaP* immune-checkpoint inhibitor-associated pneumonitis, *ICI* immune-checkpoint inhibitor, *ILD* interstitial lung disease, *BALF* bronchoalveolar lavage fluid

*p < 0.05, adjusted for multiple comparisons using the Bonferroni correction method

Expectedly, absolute number of lymphocytes in BALF showed an overall significant difference (*p* = 0.001). Paired comparison with ICIaP revealed significantly elevated lymphocyte count compared to lung cancer (*p* = 0.001) and to healthy control (*p* = 0.025), but not compared to ILD other than ICIaP (*p* = 0.210). In serum, only C-reactive protein (CRP) showed a significant difference between groups (*p* = 0.002). CRP in patients with ICIaP was significantly higher compared to patients with lung cancer (*p* = 0.001) but not to healthy controls or patients with ILD other than ICIaP (*p* = 0.291 and *p* = 0.057, respectively). Analysis of cytokines in serum and BALF are presented in Tables 5 and 6, respectively.

**Table 5:** Analysis of cytokines in serum

	ICIaP	Healthy control	ILD	Lung cancer	*p* value
IFN-γ [pg/ml]	11.4 (2.7–18.5	4.9 (1.8–9.4)	15.3 (6.5–33.9)	20.3 (8.1–44-4)	0.310
IL-1b [pg/ml]	0.5 (0.2–0.8)	0.2 (0.1–0.6)	2.4 (0.6–5.8)	3.1 (1.9–3.9)	< 0.001*
IL-2 [pg/ml]	1.9 (0.9–4.2)	1.4 (1.1–1.9)	3.3 (1.7–7.8)	5.8 (2.1–6.5)	0.018*
IL-4 [pg/ml]	9.0 (4.0–14.7)	6.4 (2.2–11.6)	27.8 (14.7–81.8)	42.0 (22.6–51.4)	< 0.001*
IL-5 [pg/ml]	3.8 (1.8–5.2)	3.1 (1.3–5.7)	4.2 (1.7–7.3)	3.4 (1.4–5.3)	0.972
IL-6 [pg/ml]	5.8 (1.8–11.1)	3.5 (1.8–7.1)	6.3 (3.2–10.2)	5.8 (3.8–8.5)	0.404
IL-8 [pg/ml]	9.4 (7.8–12.8)	7.1 (6.5–10.3)	14.7 (6.9–35.2)	16.2 (11.5–29.3)	0.068
IL-12p70 [pg/ml]	2.9 (0.6–6.2)	2.2 (1.0–4.6)	3.7 (1.3–8.5)	3.5 (2.7–8.0)	0.235
IL-13 [pg/ml]	5.5 (2.2–10.1)	3.7 (1.2–7.6)	4.3 (2.0–20.0)	9.6 (1.7–12.9)	0.482
IL-17A [pg/ml]	3.5 (0.7–12.9)	2.4 (0.6–8.6)	15.2 (6.1–31.7)	18.4 (11.1–43.1)	0.010*
TNF-α [pg/ml]	5.9 (4.5–11.1)	7.0 (4.6–8.9)	13.8 (10.0–18.6)	12.5 10.7–19.0)	< 0.001*

Data are presented as median (IQR)

*ICI* immune-checkpoint inhibitor, *ILD* interstitial lung disease, *IFN-γ* Interferon gamma, *IL* interleukin, *TNF-α* tumor necrosis factor alpha

*p < 0.05, adjusted for multiple comparisons using the Bonferroni correction method

**Table 6:** Analysis of cytokines in BALF

	ICIaP	Healthy control	ILD	Lung cancer	*p* value
IFN-γ [pg/ml]	0.8 (0.0–1.3)	0.1 (0.0–1.2)	0.0 (0.0–0.1)	0.1 (0.0–0.2)	0.035*
IL-1b [pg/ml]	0.2 (0.1–0.3)	0.2 (0.1–0.8)	0.3 (0.3–0.5)	0.3 (0.3–0.5)	0.050
IL-2 [pg/ml]	0.4 (0.3–0.8)	0.6 (0.3–1.3)	1.0 (0.5–1.6)	1.0 (0.8–1.8)	0.060
IL-4 [pg/ml]	0.0 (0.0–0.4)	0.0 (0.0–0.3)	0.0 (0.0–0.0)	0.0 (0.0–0.0)	0.074
IL-5 [pg/ml]	0.4 (0.0–0.8)	0.2 (0.0–0.6)	0.2 (0.1–0.3)	0.2 (0.1–0.3)	0.519
IL-6 [pg/ml]	126.0 (14.6–248.9)	1.9 (0.5–4.5)	0.8 (0.5–1.5)	1.5 (0.7–7.8)	0.031*
IL-8 [pg/ml]	40.4 (18.5–77.5)	39.0 (9.2–68.9)	47.5 (24.8–52.7)	56.0 (32.9–272.4)	0.838
IL-12p70 [pg/ml]	0.5 (0.5–0.5)	0.5 (0.5–0.5)	0.5 (0.5–0.5)	0.0 (0.0–0.1)	0.767
IL-13 [pg/ml]	0.0 (0.0–0.2)	0.3 (0.0–0.8)	0.0 (0.0–0.2)	0.1 (0.0–0.2)	0.542
IL-17A [pg/ml]	0.5 (0.5–0.7)	0.7 (0.3–0.7)	0.2 (0.2–0.2)	0.2 (0.2–0.2)	0.001*
TNF-α [pg/ml]	3.0 (0.8–8.8)	0.8 (0.6–2.1)	0.4 (0.1–1.0)	0.5 (0.2–1.1)	0.157

Data are presented as median (IQR)

*ICI* immune-checkpoint inhibitor, *ILD* interstitial lung disease, *IFN-γ* Interferon gamma, *IL* interleukin, *TNF-α* tumor necrosis factor alpha

*p < 0.05, adjusted for multiple comparisons using the Bonferroni correction method

From all investigated cytokines in serum, the following five cytokines were presented with an overall significant difference: IL-1b, IL-2, IL-4, IL-17A and TNF-α. However, none of the control groups showed a significant association after pairwise comparison to patients with ICIaP.

**Fig. 2:**
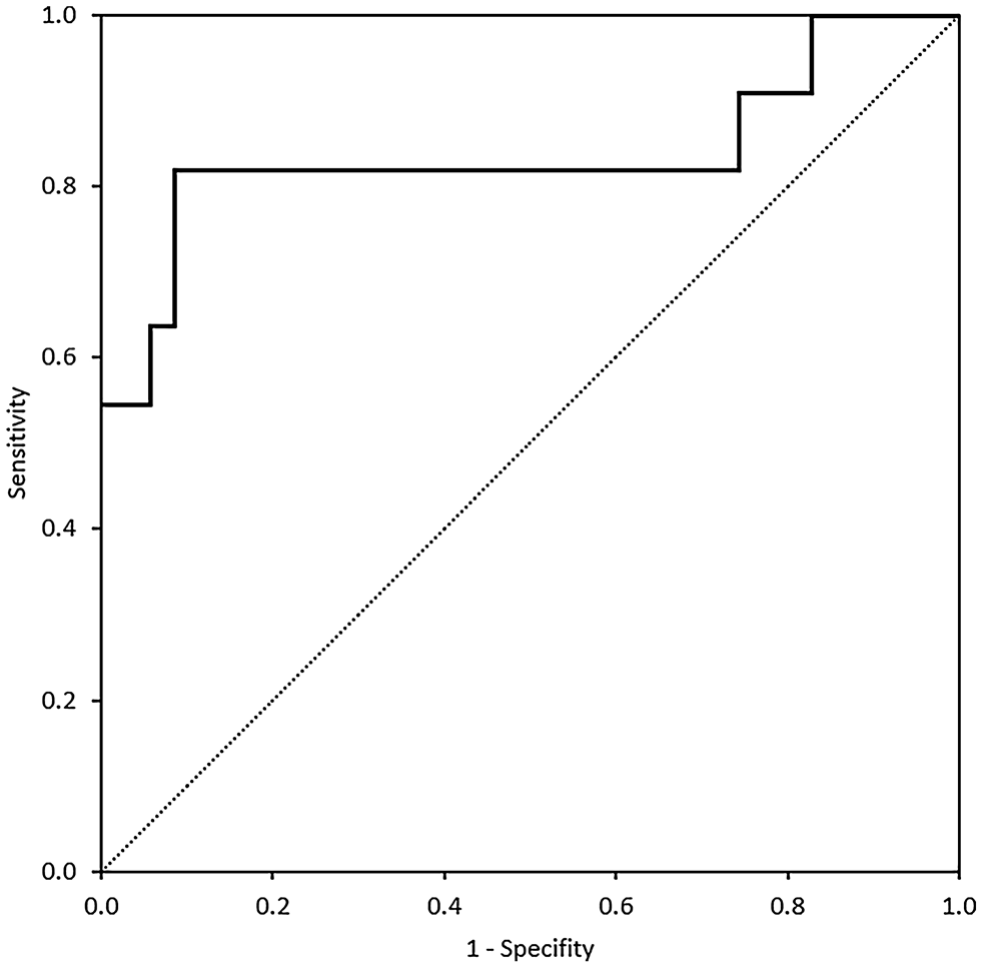
Receiver Operating Characteristic Curve for IL-6 in BALF [pg/ml]. Area Under Curve 0.836. For cut-off point of 11.8 pg/ml, sensitivity was 81.8% and specificity 91.4%

Analysis of cytokines in BALF showed an overall significance (*p* = 0.035) for IFN-γ and (*p* = 0.001) for IL-17A. Pairwise comparison unfolded a significant increase of IL-17A in patients with ICIaP compared to lung cancer (*p* = 0.011) and to ILD other than ICIaP (0.004) but not to healthy controls (*p* = 1.000), whereas IFN-γ showed no significance in pairwise comparison with ICIaP. Furthermore, cytokines in BALF revealed an overall significant increase (*p* = 0.031) of IL-6 in ICIaP as well as in paired comparison with all control groups (Fig. 1). A receiver operating characteristic curve (Fig. 2) for IL-6 in BALF correlated strongly with diagnosis of ICIaP (area under curve 0.836 (95% CI 0.657–1.0, *p* = 0.001)). Cut-off point for IL-6 equaling 11.8 pg/ml resulted in sensitivity = 81.8% and specificity = 91.4%.

## Discussion

After introducing ICIs in modern cancer treatment regimens, a new entity of irAEs has emerged including ICIaP (Chuzi et al. 2017). Since the immunological mechanism is not fully understood and, thus, diagnosis of ICIaP is not straight forward due to several differential diagnoses, the focus of this study was to assess cytokine profile in BALF and serum of patients with ICIaP compared to matched control groups consisting of healthy individuals, patients with lung cancer and patients with ILD other than ICIaP.

Overall, healthy control group revealed significantly higher recovery rate of BALF compared to other groups, which also coincides with the lowest pack years of all groups. Since pulmonary pathologies were absent in healthy subjects, bronchial collapse was much rarer than in other groups, resulting in the highest recovery of instilled saline solution for BAL. Furthermore, we found that in BALF of patients with ICIaP there was a significantly increased expression of IL-6, that could be confirmed after pairwise comparison to all control groups. Similarly, IL-17A was significantly increased in patients with ICIaP compared to lung cancer and ILD, but not compared to healthy controls. Additionally, IFN-γ in BALF was overall significantly increased but not for any pairwise comparison with ICIaP. In serum, we were not able to identify significantly increased cytokines in patients with ICIaP.

Expressed by Th2 cells, monocytes, macrophages, dendritic cells and bone marrow stroma, IL-6 is an important cytokine for vast inflammatory responses including induction of acute phase proteins (APP) and enhancement of T-cell proliferation as well as polarization of Th17 cells. Th17 cells are a subset of CD4 cells known to trigger massive inflammatory diseases with a tendency for autoimmune reactions (Delves et al. 2017). Th17 cells reside mostly in tissues exposed to the external environment such as gastrointestinal tract, skin, and respiratory tract, and express themselves cytokines, notably IL-17A, IL-21 and IL-22. Both, autoimmune diseases and chronic inflammatory disorders of the lung are suspected to arise from dysregulation of Th17 cells such as chronic obstructive pulmonary disease (COPD), bronchial asthma, rheumatoid arthritis (RA), chronic hypersensitivity pneumonitis and other forms of interstitial fibrosis (Eyerich et al. 2017; Nembrini et al. 2009; Weaver et al. 2013; Zhang et al. 2019). In addition, in a mice model, recovery from acute lung injury in absence of Th17 cells was faster compared to those with Th17-positive cellular immune reaction (Wang et al. 2018). It seems plausible that Th17 cells stimulated by IL-6 play an important role in the still vague pathogenesis of ICIaP.

Although IL-6 levels in BALF of the ICIaP group were significantly increased compared to all control groups in our study, their origin remains uncertain. According to a recent study by our group, IL-6 was not significantly increased in BALF of patients with untreated lung cancer and other lung diseases (sarcoidosis, ILD), deeming IL-6 appropriate as biomarker of unspecific inflammation (Bezel et al. 2021). These findings correspond to the results of our study, where solely ICIaP showed elevated levels of IL-6. Therefore, increased IL-6 levels in our study could originate from inflammation due to ICIaP. However, enhanced and polarized through IL-6 expression, Th17 cells may serve as a more precise biomarker. Determining Th17-cell count, IL-21, IL-22 and subgroups of IL-17 including IL-17A in BALF and serum, respectively, might be indispensable to gain further knowledge about their role in immune-related pneumonitis. At least, there is one study showing significant association of elevated levels of IL-17A in BALF and serum of patients with ICIaP (Wang et al. 2020). The results from our study partially support the findings from the aforementioned study, as we also detected elevated levels of IL-17A in BALF of ICIaP that proved to be significantly higher compared to patients with lung cancer and patients with ILD other than ICIaP, but not in comparison to healthy subjects.

Owed to the broad inflammatory response of IL-6, there is little use for IL-6 as single diagnostic tool. However, as important element of pathogenesis in several diseases, antibodies against IL-6 might be used as therapeutic approach. Initially used for treatment of RA, the anti-IL-6R antibody Tocilizumab has since been successfully approved for therapy of systemic juvenile idiopathic arthritis and polyarticular juvenile idiopathic arthritis (Sheppard et al. 2017). Due to the importance of IL-6 in immune response, Tocilizumab may have the potential to play a similarly important role in regulation thereof, as there are numerous preclinical and clinical studies investigating further example of application such as giant cell arteritis, polymyalgia rheumatica and large vessel vasculitis (Sheppard et al. 2017). However, IL-6 is known to act ambiguous in relation to cancer, both promoting tumor growth and impeding tumor growth by immune stimulation (Fisher et al. 2014). Although some studies have shown very promising results in targeting IL-6–IL-6R axis with Tocilizumab (Esfahani et al. 2020; Kampan et al. 2018; Stroud et al. 2019), concern remains, if manipulation of IL-6 may also support tumor growth as it has been discussed in the past (Kumari et al. 2016).

There are several limitations to this study. First, a relatively small number of patients treated with ICI paired with low incidence of ICIaP resulted in a small sample size. Consequently, general validity of results is limited. Second, albeit BAL is widely accepted as valid tool for assessing the cellular composition of the alveolar compartment and, thus, for contributing to diagnosis of various lung diseases (Gharsalli et al. 2018), it presents constraints owed to dilution necessary to acquire BALF. Therefore, lower cytokine readings in BALF may go undetected, which could skew data and lead to flawed conclusions.

## Conclusion

Cytokine profile assessed through BAL shows promising potential for facilitating diagnosis and understanding of the pathophysiology of ICIaP. As such, IL-6 seems to play a relevant role, which may herald also therapeutic implications for the use of Tocilizumab. In addition, importance of Th17-cells in pathogenesis of ICIaP appears worthy of future investigations. Further studies will have to be conducted to determine indications of cytokine analysis in BALF in patients with suspected ICIaP.

## Data Availability

The datasets used and analysed during the current study are available from the corresponding author on reasonable request.

## Abbreviations

AIP: Acute interstitial pneumonia
APP: Acute phase protein
ARDS: Acute respiratory distress syndrome
BAL: Bronchoalveolar lavage
BALF: Bronchoalveolar lavage fluid
COPD: Chronic obstructive pulmonary disease
CRP: C-reactive protein
CT: Computed tomography
CTLA-4: Cytotoxic T-lymphocyte-associated antigen 4
HP: Hypersensitivity pneumonitis
ICI: Immune-checkpoint inhibitor
ICIaP: Immune-checkpoint inhibitor-associated pneumonitis
IFN-γ: Interferon gamma
IL: Interleukin
ILD: Interstitial lung disease
IQR: Interquartile range
irAE: Immune-related adverse events
mAB: Monoclonal antibodies
NSIP: Non-specific interstitial pneumonia
OP: Organizing pneumonia
PD-1: Programmed cell death protein 1
PD-L1: PD-1 ligand
RA: Rheumatoid arthritis
RB-ILD: Respiratory bronchiolitis interstitial lung disease
SD: Standard deviation
TNF-α: Tumor necrosis factor alpha
UIP: Usual interstitial pneumonia
